# Supplementary material for: Integrated Transcriptomic and Metabolomic Analyses Uncover the Differential Mechanism in Saline–Alkaline Tolerance between Indica and Japonica Rice at the Seedling Stage
Source: Int J Mol Sci. 2023 Aug 3;24(15):12387. doi: 10.3390/ijms241512387 (PMC10418499; doi:10.3390/ijms241512387)
Supplement: Supplementary file 1 [file ijms-24-12387-s001.zip › Supplementary Figures.pdf]

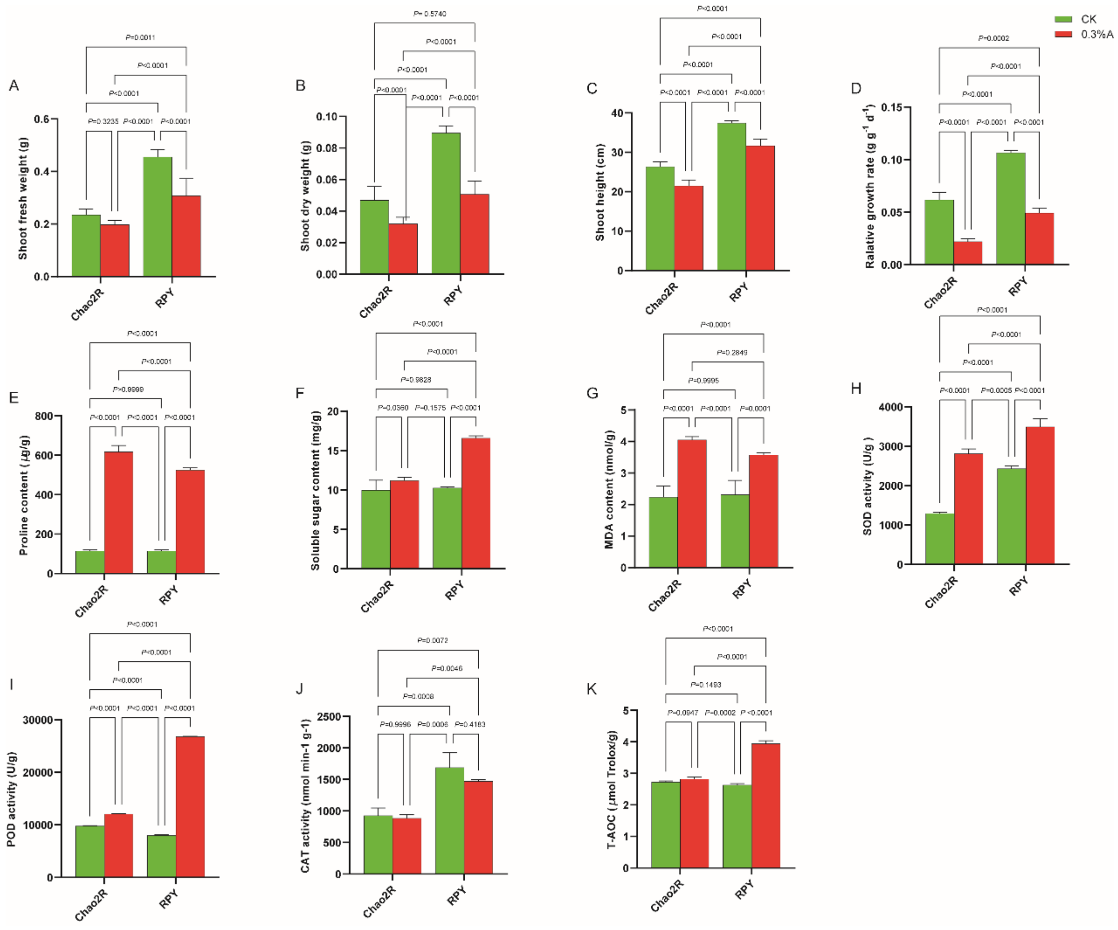

Figure S1. Phenotypical and physiological characteristics of Chao2R and RPY geng with and without the saline-alkaline condition. (**A**) shoot fresh weight. (**B**) Shoot dry weight. (**C**) Shoot height. (**D**) relative growth rate. (**E**) Proline content. (**F**) Soluble sugar content. (**G**) Malondialdehyde content. (**H**) Superoxide dismutase activity. (**I**) Peroxidase activity. (**J**) Catalase activity. (**K**) Total antioxidant capacity.

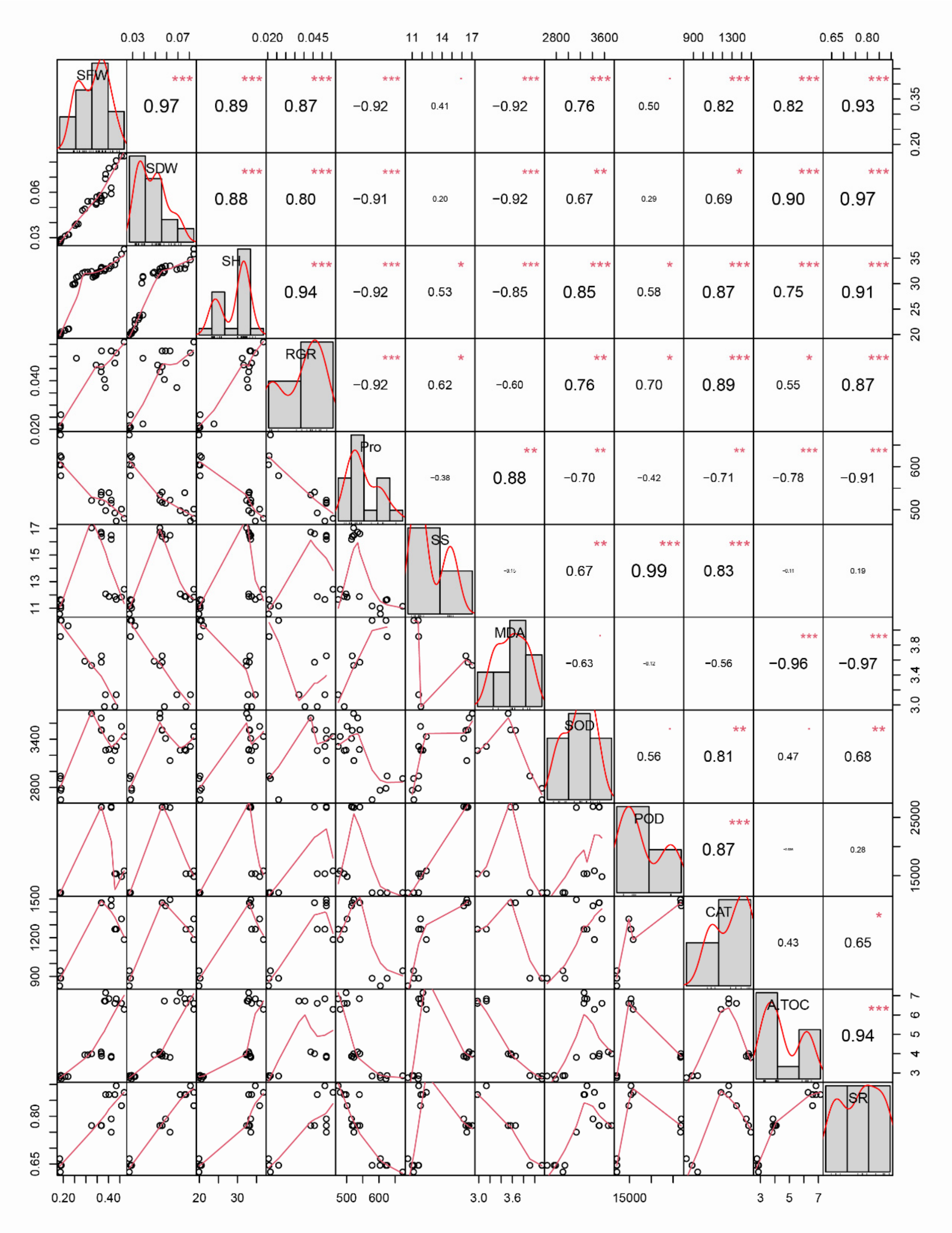

Figure S2. Pearson's correlation coefficient between phenotype and physiological traits of Chao2R and RPY seedlings with and without saline-alkaline treatment.

The lower left represents the linear regression statistics between each two traits, the middle represents the distribution of each trait, and the upper right number represents the correlation coefficient. Asterisks represent significance (**P* < 0.05; ***P* < 0.01; ****P* < 0.001). SDW: Shoot dry weight; SFW: shoot fresh weight; SH: shoot height; RGR: relative growth rate; Pro: Proline content; SS: Soluble sugar content; CAT: Catalase activity; POD: Peroxidase activity; SOD: Superoxide dismutase; MDA: Malondialdehyde content; T-AOC: Total antioxidant capacity; SR: Survival rate.

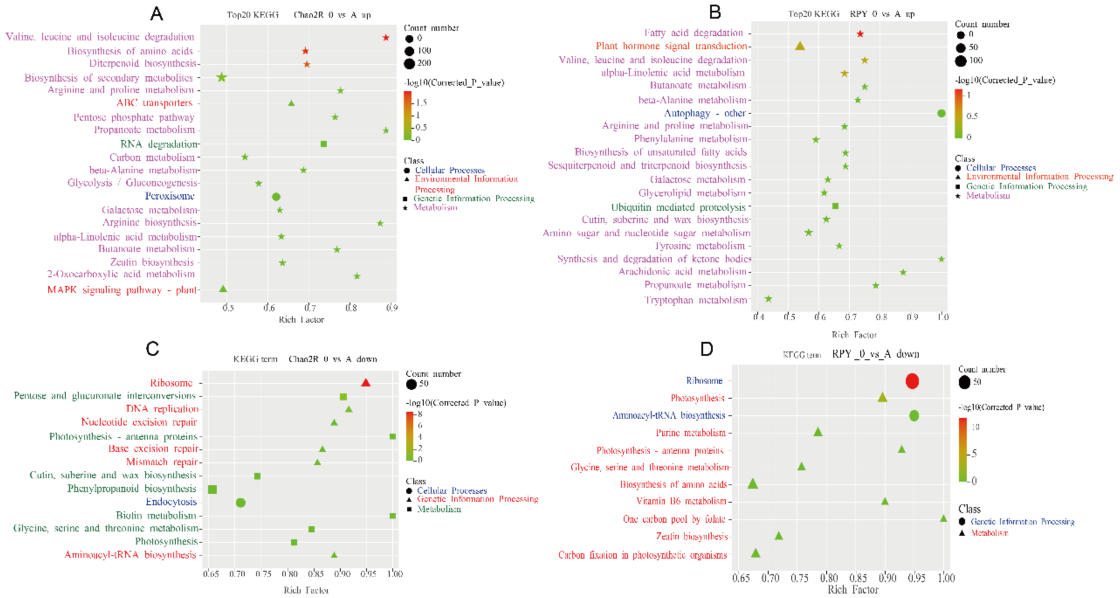

Figure S3. KEGG enrichment of DEGs under saline-alkaline treatment for 72 h compared with untreated control of Chao2R and RPY geng. Up-regulated DEGs in Chao2R (**A**), up-regulated DEGs in RPY geng (**B**), down-regulated DEGs in Chao2R (**C**), down-regulated DEGs in RPY geng (**D**). 0 and A represent the untreated control and saline-alkaline treatment for three days, respectively.

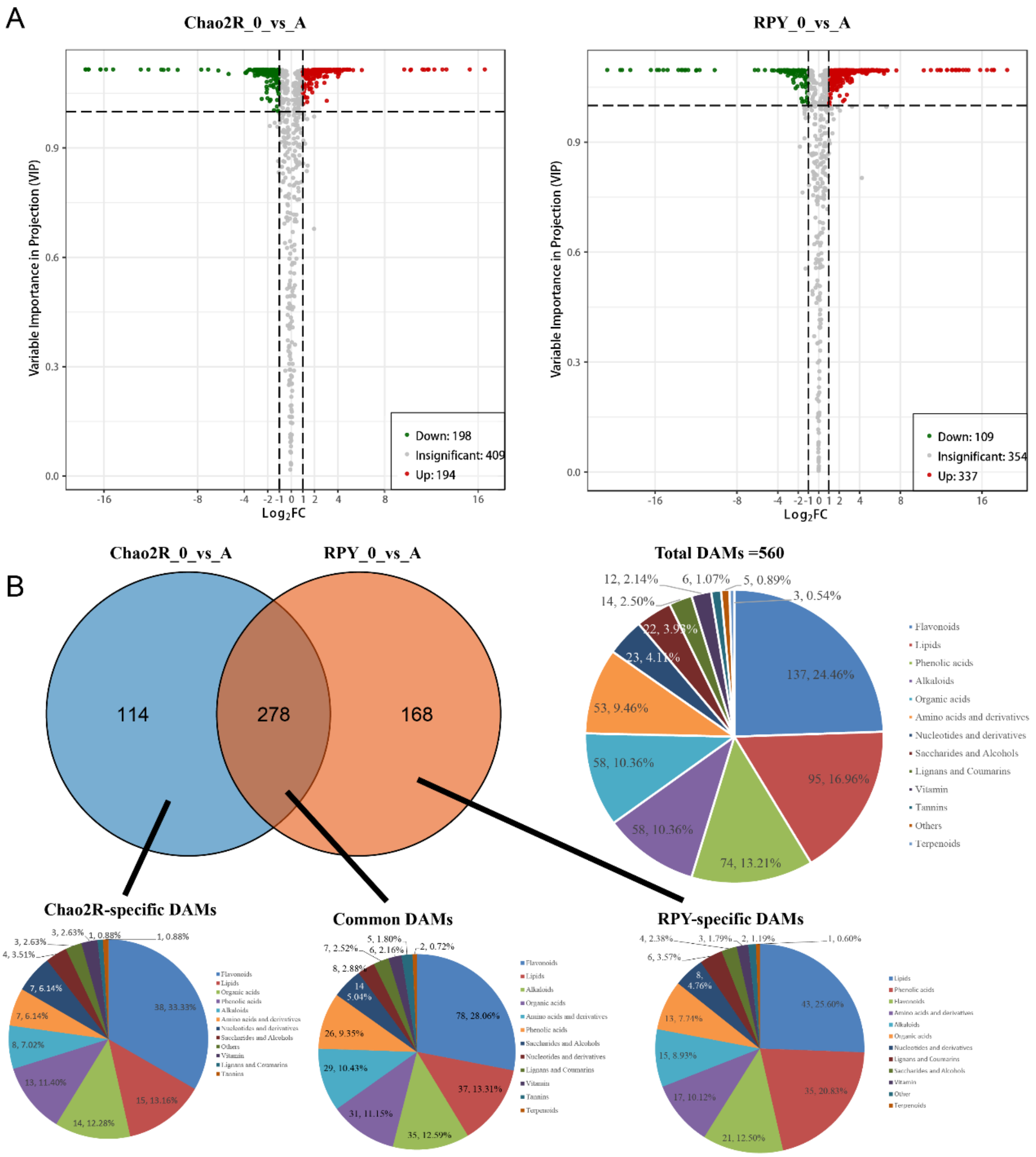

Figure S4. Comparison of the differentially accumulated metabolites (DAMs) between Chao2R and RPY geng seedlings under saline-alkaline condition. (**A**) Volcano map of DAMs in Chao2R (left) and RPY geng (right) after exposure to saline-alkaline condition compared with untreated control (short for 0_vs_A). (**B**) Venn diagram showing the DAMs (upper left) and classification of the total 560 DAMs (upper right) between both genotypes with saline-alkaline treatment compared with control condition. Classification of the specifically DAMs in Chao2R (lower left) and RPY geng (lower right) and their common DAMs (lower middle).

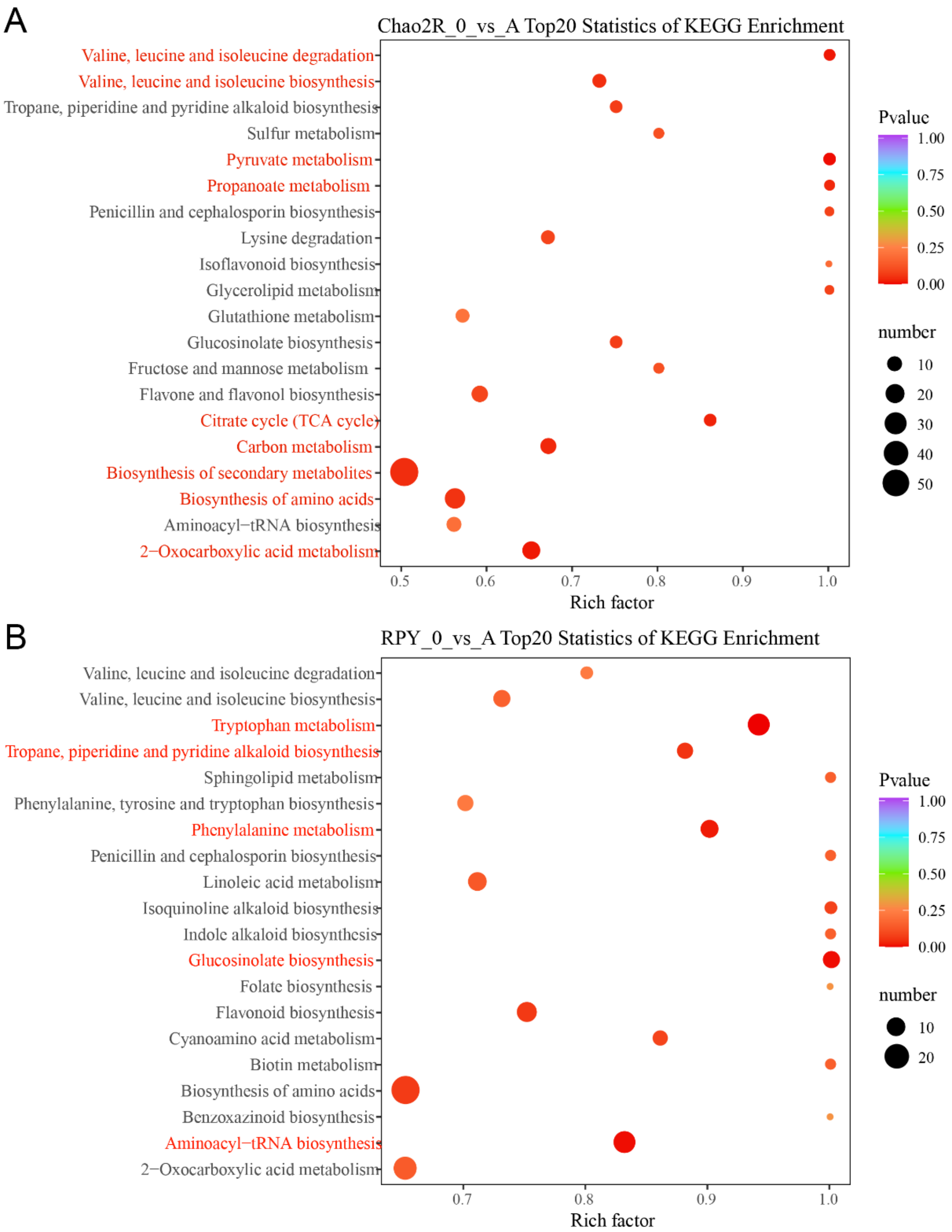

Figure S5. Top 20 enriched KEGG pathways of DAMs in Chao2R (**A**) and RPY geng (**B**) treated with saline-alkaline stress condition in contrast to untreated control. 0 and A represent the untreated control and saline-alkaline treatment, respectively. Red fonts mean the significantly enriched KEGG pathways of DAMs in Chao2R and RPY geng.

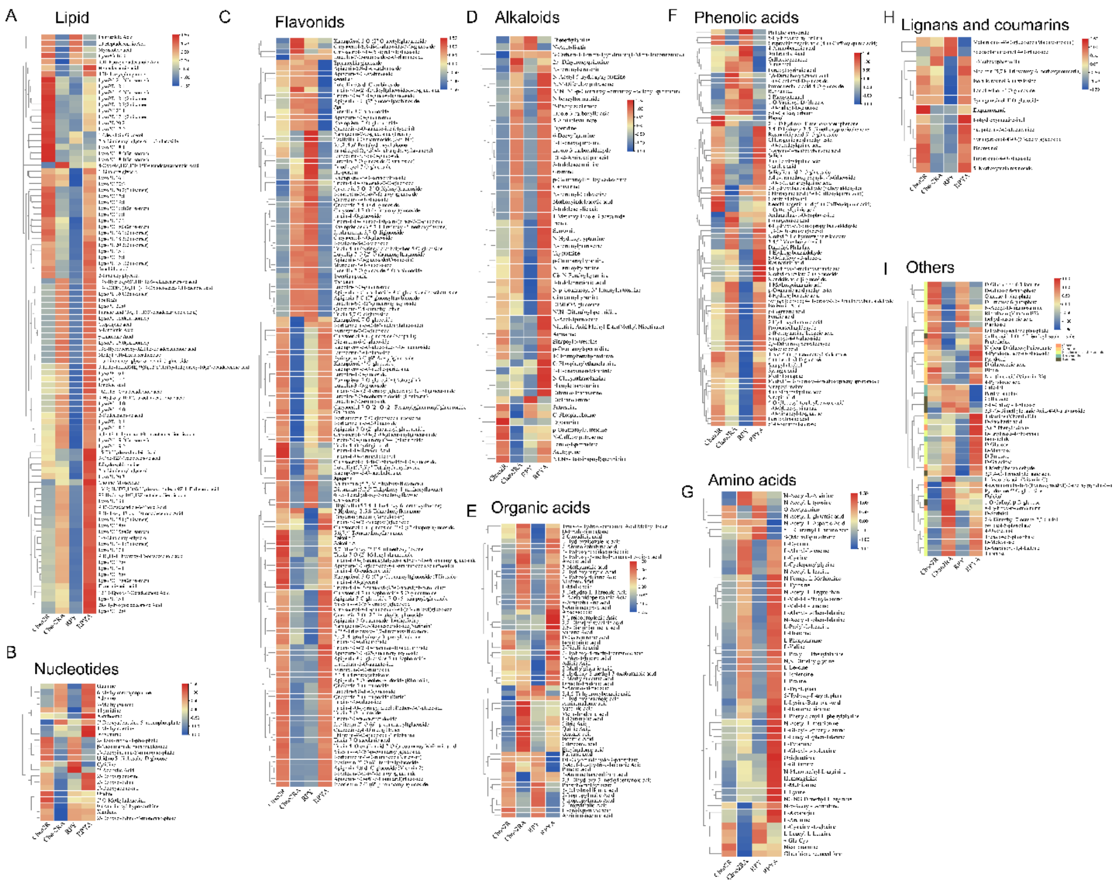

Figure S6. Heatmaps showing the 560 DAMs of Chao2R and RPY geng with saline-alkaline stress treatment compared with the untreated control. Lipids (**A**), nucleotides(**B**), flavonoids (**C**), alkaloids (**D**), organic acids (**E**), phenolic acids (**F**), amino acids (**G**), lignans and coumarins (**H**), and others (**I**) including vitamins, saccharides and alcohols, terpenoids, as well as tannins. Chao2R and RPY mean samples of Chao2R and RPY geng under normal condition, respectively; Chao2RA and RPYA mean samples of Chao2R and RPY geng treated with saline-alkaline stress for 72 h, respectively

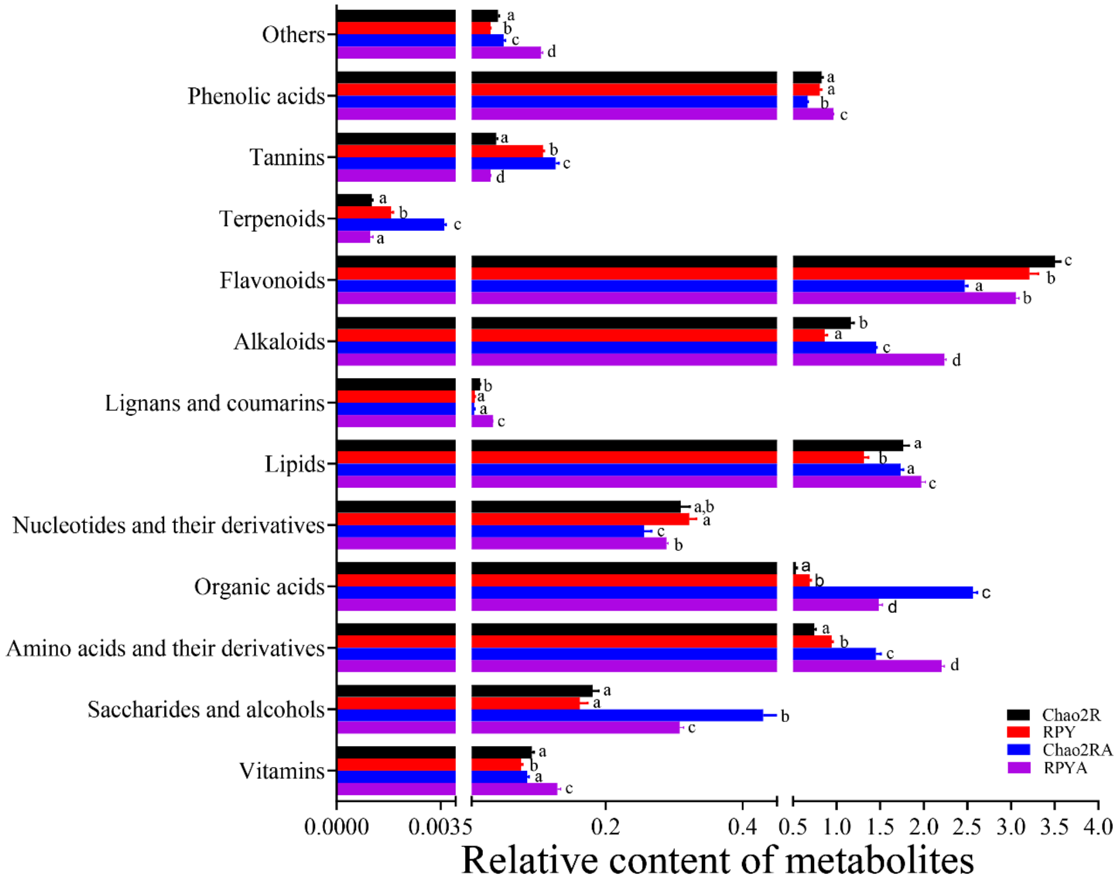

Figure S7. Relative content level of different kinds' metabolites in Chao2R and RPY geng with and without saline-alkaline treatment. Small letters indicate significantly different ($P < 0.05$) within the same metabolite. Chao2R and RPY mean samples of Chao2R and RPY geng without saline-alkaline treatment, respectively; Chao2RA and RPYA mean samples of Chao2R and RPY geng with saline-alkaline stress treatment, respectively

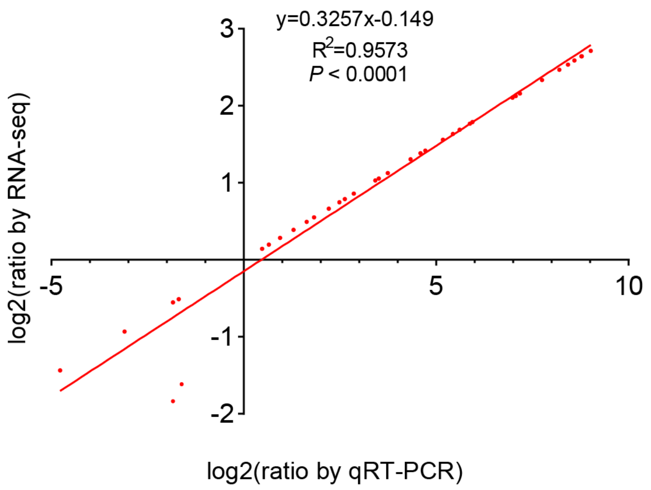

Figure S8. Correlation analysis between qRT-PCR and RNA-seq data
